# Relation of skinfold thickness with the serum lipids, glucose and blood pressure among Indian sedentary office workers

**DOI:** 10.1186/s41043-024-00706-0

**Published:** 2024-12-18

**Authors:** Nikhil Gopal Naik, Baskaran Chandrasekaran, Rakshith N. Patil, Saritha U. Kamath

**Affiliations:** 1 Department of Medical Laboratory Technology, Manipal College of Health Professions, Manipal Academy of Higher Education, Manipal, Karnataka 576104 India; 2 Department of Exercise and Sports Sciences, Manipal College of Health Professions, Manipal Academy of Higher Education, Manipal, Karnataka 576104 India; 3 Department of Orthopaedics, Kasturba Medical College, Mangalore, Manipal Academy of Higher Education, Manipal, Karnataka 575004 India

**Keywords:** Cardiovascular disease markers, Non-invasive, Relation, Fat percentage, Skinfold

## Abstract

**Background:**

Serum glucose, cholesterol, triglycerides and high-density lipoproteins (HDL) are established cardiovascular disease (CVD) markers, however accessibility to these markers is less in individuals from low-middle income countries. The non-invasive CVD risk marker especially skinfold measured fat percentages are less explored for its relevance with established serum biochemistry markers.

**Methods:**

A cross-sectional study was conducted in 70 sedentary office workers (aged 30–40 years) who were healthy. Peripheral fat percentages were estimated from four skinfold thickness measurements and biochemistry markers were measured and analysed using standard laboratory measurements. Blood pressure was also measured. Multivariate linear regression models were drawn to establish the association between the non-invasive and invasive CVD risk markers.

**Results:**

The skinfold measured fat percentage was negatively associated with the HDL (coefficient β = -0.15, standard error SE = 0.07, *p* < 0.05). No significant relation between the other biochemistry parameters with the skinfold thickness. Age and BMI were found to be mediating the above relationship.

**Conclusions:**

Skinfold thickness derived fat percentage is associated with the few of the CVD markers (especially HDL). Age and BMI are crucial mediating factors for the fat measurement. Skinfold measurements could be included as part of routine primary care screening for CVD risk, alongside invasive biochemistry parameters.

## Introduction

Cardiovascular diseases (CVDs) are a leading cause of morbidity and mortality worldwide, with a particularly high burden in low- and middle-income countries (LMICs), including India [1]. According to the World Health Organisation, approximately 80% of the 20.5 million CVD related deaths occur in LMICs [1]. Rapid urbanization, sedentary lifestyles, and dietary changes have driven the surge in the CVD risk factors, making early detection and prevention critical in India [2]. To address this growing public health challenge, biochemistry parameters such as lipid profiles, glucose levels, and inflammatory markers have emerged as essential tools for identifying individuals at heightened risk of developing cardiometabolic diseases [3]. As these parameters mark the atherogenic risk, vascular dysfunction, increased blood pressure (BP) and eventually CVD risk, knowledge on these risk factors are crucial in diagnosis and understanding the CVD risk [4].

Numerous body composition measures (anthropometry, bioelectric impedance, dual energy x-ray absorptiometry, computerised tomography or ultrasound) are available to quantify fat patterns and distribution, which are the sub-ordinate measures of CVD risk, but differ in terms of accuracy, availability and cost [5]. Though inexpensive and widely used, traditional anthropometric measures such as body mass index (BMI) provide a generalized assessment of obesity but often fail to capture fat distribution, especially in the peripheral and central regions [6]. Skinfold thickness, a non-invasive and easily measurable parameter, offers a more precise evaluation of subcutaneous fat [7, 8]. When coupled with skinfold measured body fat percentages, it allows for the prediction of cardiometabolic risk with greater specificity than traditional BMI and waist-hip ratio [9]. Observational studies have established strong associations between skinfold thickness and key biochemistry parameters such as lipid profiles and glucose levels in community adults from Peru [10] and India [11], underscoring the importance of localized fat accumulation in metabolic disturbances.

For LMICs like India, where healthcare resources can be limited, non-invasive and cost-effective screening tools such as skinfold thickness measurements become particularly valuable for measuring CVD risk. These markers are easy to administer in field settings, require minimal equipment, and offer immediate insights into an individual's health status. Given the high prevalence of sedentary lifestyles and associated CVD risk among office workers in India [11], there is an urgent need to implement robust quality measures to assess risks, driven by a strong desire to make a meaningful impact, which serves as a powerful incentive to apply principles of change [12].

The present study aimed to explore the relationship of skinfold thickness derived fat percentages with biochemistry parameters (fasting glucose and lipid profile) and blood pressure (BP) among Indian sedentary office workers. We hypothesize that higher estimated fat percentages, measured via peripheral skinfold thickness, are associated with adverse cardiometabolic profiles, as indicated by elevated biochemistry parameters (elevated lipid and glucose profiles) and arterial pressure, thus offering a reliable non-invasive screening tool for this sedentary Indian office workers.

## Methods

### Study design and participants

This cross-sectional study was conducted among sedentary office workers in a multifaceted university of coastal region of Karnataka, India, from December 2022 to April 2023. The study aimed to examine the association between estimated body fat percentages, as measured by peripheral skinfold thickness, and key biochemistry parameters indicative of CVD risk. After institutional ethical committee approval and prospective trial registration, the managers of fifteen administrative offices with desk-based jobs of a multifaceted university in coastal Karnataka was contacted for potential participants. To be included, the participants should be: (1) desk-based office workers with at least six hours of computer engagement for their work; (2) full time > 35 h of work engagement in a week; (3) with a minimum of six months of employment in the current job. Office workers were excluded if self-reported weekly physical activity engagement was 30 min daily for at least 5 days a week, in the past three months, with self-reported cardiovascular or metabolic conditions limiting the participation of physical activity, pregnant and recent (< 2 weeks) hospitalisation for chronic diseases. Participants undergoing weight loss treatment were also excluded. After screening for eligibility, participants were informed about the study objectives and test procedures and instructed to report for laboratory investigations and skinfold measurements within one week of recruitment. On the day of their appointment, participants were required to have fasted for at least 8 h and to have had at least 8 h of sleep prior to the measurement of skinfolds and biochemical parameters.

### Sample size estimation

Using Z test of correlation: tetrachoric model, a sample size of 64 was required to establish a moderate correlation (H1 corr ρ = 0.5) of triglycerides with skinfold derived fat percentages at a α err prob of 0.05 and power of 80% (G*Power version 3.1.9.6, University of Kiel, Germany). A total of 70 participants, aged 30–50 years, were recruited from the administrative offices of fifteen academic institutions of a multifaceted university of coastal Karnataka, India.

### Ethical considerations

The study was approved by the Institutional Ethics Committee of Kasturba Medical College and Kasturba Hospitals (IEC2:388/2022) and prospectively registered in Clinical Trials Registry of India (CTRI/2023/03/050465). Informed consent was obtained from all participants prior to data collection, ensuring adherence to ethical principles outlined in the Declaration of Helsinki. Appropriate written & informed consent was obtained from all the included participants.

### Data collection

#### Skinfold measured fat percentages and body mass index

The peripheral fat percentages were estimated from the four skinfold at different sites of the body (infrascapular, triceps, suprailliac and thigh) using a skinfold calliper (Lange, Beta Technology Inc., Cambridge, MA). A postgraduate in exercise and sports science administered the skinfold measurements using the Lange calliper and standards outlined by the developer and the International Society for the Advancement of Kinanthropometry [8]. Skinfold thickness at four sites was measured twice using callipers. If the two values were within 2 mm of each other, the higher value was recorded. If the difference exceeded this threshold, a third measurement was taken to ensure accuracy. The body density was estimated using gender specific regression equations from the sum of the skinfold measurements outlined by Petersen, 2003 [8]. Body fat percentages were estimated from the body density using Siri’s equation [13]. The root means square error, related to interrater reliability, for the calculation of subcutaneous BF% has been found to be 4.6% [8].

Weight and height were measured using calibrated weighing scale and stadiometer without shoes to nearest 0.1 kg and 0.1 cm respectively [11]. Body mass index was estimated using the formula BMI = weight (kg)/ height (m^2^) [14].

#### Biochemistry parameters

Blood samples were collected after an overnight fast of at least 8 h at the participant’s respective workplaces by a postgraduate in medical laboratory technology. Serum glucose, total cholesterol, high-density lipoprotein (HDL) and triglycerides, were measured using enzymatic kit assay method using spectrophotometer [14]. Samples were analyzed at an accredited central lab following quality control protocols. Following cut points were taken as threshold: triglycerides ≥ 150 mg/dl; cholesterol ≥ 200 mg/dl; HDL ≤ 60 mg/dl; and serum glucose ≥ 126 mg/dl for optimum cardiometabolic health [14].

#### Blood pressure

Blood pressure was measured as per the standard guidelines laid by the European Health Examination Survey standards [15]. The participants were instructed to sit comfortably in an armchair with their feet flat on the floor. The primary author measured blood pressure three times using a calibrated sphygmomanometer (Diamond, India), and the highest reading was used for the final analysis.

### Outcome variables and covariates

The primary outcome variables included the following: body fat percentages estimated from body density and the sum of peripheral skinfold thickness (triceps, subscapular, suprailiac, midthigh) as outlined by the Peterson 2003 [8]. The main exposure variable was lab assessed serum biochemistry parameters: fasting glucose, total cholesterol, HDL and triglycerides. Covariates included age, sex and BMI assessed during initial screening.

### Statistical analysis

All statistical analyses were performed using Jeffrey’s Amazing Software Package version 0.19.1.0 [JASP team, University of Amsterdam] [16]. A two-sided p-value < 0.05 was considered statistically significant. The distribution of biochemistry parameters and skinfold measurements was assessed for normality using the Shapiro–Wilk test. As non-normalised, the participant characteristics were summarized using median and interquartile range for continuous variables (skinfold derived fat percentages, serum biochemistry parameters, BMI and blood pressure) and frequencies (percentages) for categorical variables (gender). Multivariate Linear regression models were employed to examine the association between each biochemistry parameter (independent variable) and skinfold derived body fat percentage (dependent variable) and, adjusting for potential confounders such as age, gender and BMI. The results were expressed as beta coefficients (β) with 95% confidence intervals (CI). To account for potential measurement errors in skinfold thickness, we conducted sensitivity analyses by excluding outliers and adjusting for different skinfold sites. The variance inflation factor (VIF) was calculated to assess multicollinearity in the regression models. A VIF < 5 was considered acceptable. Further the results of the association between the variables were visualised using the scatter plots and the path diagrams for strength and extent of the association between the serum biochemistry parameters (cholesterol, triglycerides, HDL and fasting glucose) and the skinfold measured fat percentages. Mediation analyses were performed to evaluate causality of the latent variables (age, gender and BMI) affecting the relation between the serum biochemistry parameters and the skinfold derived fat percentages.

## Results

**Fig. 1:**
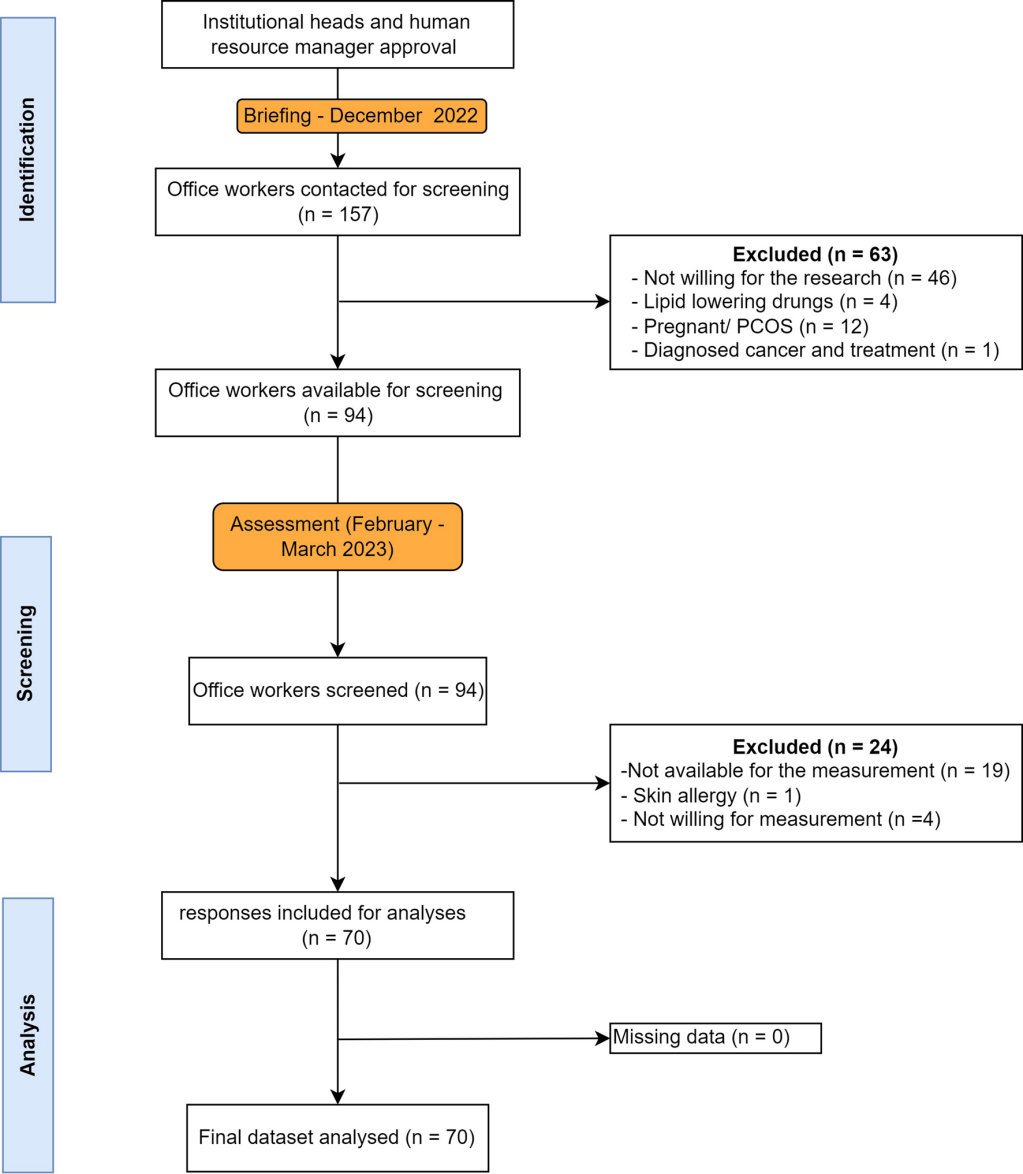
Participants screening and inclusion

Of the 157 office workers were available for the screening, only 70 (45%) completed the screening procedures (skinfold and biochemistry parameters measurement). The major exclusion criteria were ‘not willing for the measurement’ (n = 55, 35%). The flow and the included participants were illustrated as a flowchart (Fig. 1).

### Baseline characteristics

Table 1 depicts the baseline characteristics of the participants participated in the study. Majority of the participants were female and young age. Male office workers had high peripheral fat compared to the optimal threshold. Almost all the participants had a normal mean arterial pressure and lipid profile except high density lipoproteins (HDL < 60 mg/dl). Majority of the participants had a low HDL (median = 37.39 mg/dl, 95% confidence interval = 32.93, 44.98).

**Table 1:** Baseline characteristics of the participants

Characteristics	Overall^#^ (n = 70)	Men^#^ (n = 28)	Women^#^ (n = 42)
*Demographics*			
Age (years)	36 (32, 42)	36.5 (33.50, 36.50)	35.50 (31.00, 35.50)
Weight (kgs)	68.49 (60.43, 77.73)	70.57 (62.19, 77.47)	66.53 (58.54, 77.73)
BMI (kg/m2)	24.74 (22.33, 27.22)	25.21 (22.52, 27.81)	24.64 (22.10, 26.99)
*Blood pressure*			
SBP (mmHg)	120 (112, 126)	122 (112, 128)	120 (108, 126)
DBP (mmHg)	80 (76, 84)	82 (78, 86)	79 (76, 84)
MAP (mmHg)	93.33 (89.50, 97.33)	93.67 (92, 98.67)	93.33 (87, 97.33)
*Biochemistry parameters*			
Total cholesterol (mg/dl)	179.66 (145.85, 197.26)	169.76 (138.04, 191.72)	184.62 (153.66, 198)
Fasting glucose (mg/dl)	97.64 (93.12, 108.07)	99.20 (93.57, 108.88)	96.01 (91.24, 108.07)
HDL (mg/dl)	37.39 (32.93, 44.98)	33.89 (30.62, 43.32)	37.96 (33.32, 45.24)
Triglycerides (mg/dl)	114.29 (94.48, 136.98)	126.14 (93.15, 143.56)	112.65 (97.75, 131.42)
*Skinfold measurement*			
Fat (%)	23.25 (20.46, 31.21)	30.66 (29.03, 32.71)	21.27 (19.06, 23.06)
Fat (kgs)	20.29 (14.91, 24.77)	23.39 (20.88, 28.57)	16.46 (13.31, 21.95)

^#^expressed as median (interquartile range)

BMI: body mass index, DBP: diastolic blood pressure, HDL: high density lipoproteins, MAP: mean arterial pressure, SBP: systolic blood pressure

### Relation of skinfold-based fat percentage with biochemistry parameters

**Fig. 2:**
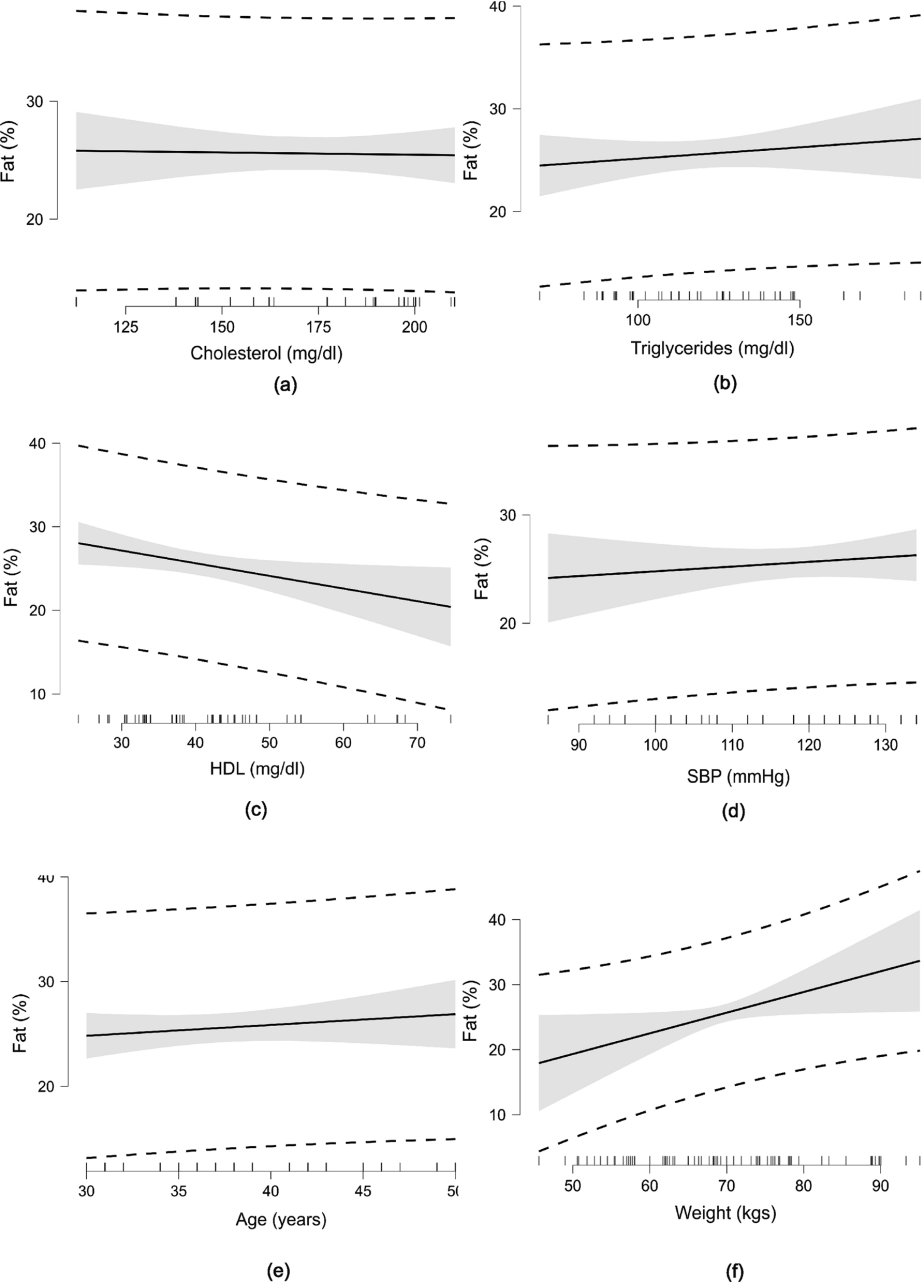
Relation of skinfold measured fat percentage with biochemical parameters and other latent variables

Null model included cholesterol, HDL, triglycerides, fasting glucose, SBP, DBP and MAP (R^2^ = 0.45, adjusted R^2^ = 0.35, *p* < 0.001). The skinfold measured fat percentage was negatively associated with the HDL (β = -0.15, standard error SE = 0.07, *p* < 0.05). However, when the other mediating factors (age, gender and BMI) added to the null model, the above association of skinfold measured fat percentages with the HDL turned insignificant. Other biochemistry and blood pressure variables were not significantly associated with the skinfold measured fat percentages. Table 2 shows the association of the biochemistry and blood pressure variables with the skinfold measured fat percentages. Figure 2 shows the association between independent variables of the biochemistry and blood pressure with the skinfold measured fat percentages.

**Table 2:** Relation of skinfold-based fat percentage with biochemistry parameters

Characteristics	Unadjusted model^†^			Adjusted model^†#^		
	Estimate (β)^§^	SE	*P* value	Estimate (β)^§^	SE	*P* value
*Biochemistry parameters*						
Total cholesterol (mg/dl)	0.01	0.03	0.811	0.03	0.02	0.174
Fasting glucose (mg/dl)	0.10	0.07	0.134	0.08	0.06	0.192
HDL (mg/dl)	** − 0.15**	**0.07**	0.025*	− 0.07	0.06	0.252
Triglycerides (mg/dl)	0.01	0.02	0.847	− 0.02	0.03	0.474
*Blood pressure*						
SBP (mmHg)	52.34	89.20	0.560	30.26	75.12	0.688
DBP (mmHg)	104.55	178.36	0.560	60.40	150.21	0.689
MAP (mmHg)	156.86	266.57	0.560	90.72	225.34	0.689

^†^analysed using multivariate regression models; ^#^adjusted for age, BMI and gender

^§^β denotes correlation coefficient, *p < 0.05

BMI: body mass index, DBP: diastolic blood pressure, HDL: high density lipoproteins, MAP: mean arterial pressure, SBP: systolic blood pressure, SE: standard error

### Mediation analyses

**Fig. 3:**
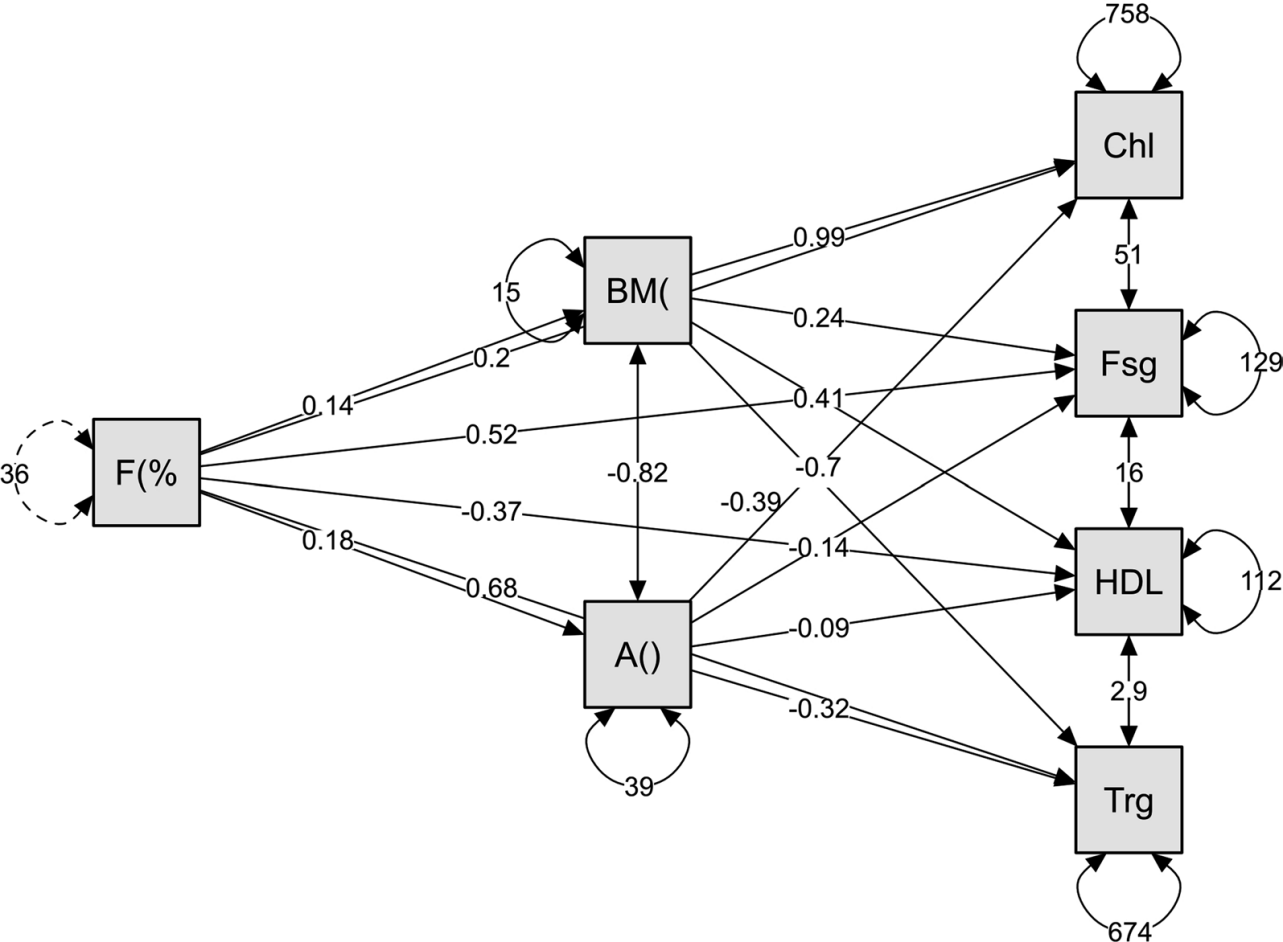
Mediation analyses of age and body mass index influencing the association between the skinfold measured fat percentage and biochemistry parameters. Chl–cholesterol, Fsg–fasting glucose, HDL–high density lipoprotein, Trg–triglycerides, BM(-body mass index (kg/m2), A( )–age (years), F(%-fat (%)

Among the proposed latent variables, the age and BMI were found to significantly mediating the association between the skinfold measured fat percentages and the biochemistry variables. However, there is a negative association between age and BMI with significantly influencing the association between the skinfold measured fat percentages and the HDL (Fig. 3).

## Discussion

The present cross-sectional study explored the potential association between the skinfold measured fat percentages and the serum biochemistry parameters in sedentary office workers. Among all the serum biochemistry biomarkers, only HDL was observed to be negatively associated with the skinfold thickness (estimated fat percentages). Though positive association was observed between the fat percentages and other lipid variables of biochemistry which are already known positive cardiometabolic disease risk markers, the association are not statistically significant. The age and BMI are identified to be the significant mediating factors to both skinfold derived fat percentage and the biochemistry risk markers.

Our study found one unit increase in HDL was associated with -0.15 units of skinfold fat percentages. Our findings concur with the previous observational studies that has concluded that higher HDL and lower cholesterol, triglycerides associated favourably with lower skinfold thickness derived fat percentages [17, 18]. HDL involves in reversal cholesterol transport with receiving free cholesterol and phospholipids from the chylomicrons and peripheral tissues and return to liver for excretion. Convincing evidence now exists to conclude the beneficial effects of HDL on the vascular function and protection against atherosclerotic cardiovascular diseases [19, 20]. Hence any increase in HDL cholesterol is speculated to be associated with the favourable reduction in skinfold derived fat percentages. Our study findings confirm the above notion. Shi et al. 2024 concluded that triceps skinfold thickness was associated with increased cardiometabolic diseases in 9440 Chinese participants of China Health and Nutrition Survey [21]. Similarly, Mitu et al. 2022 also claimed that lower HDL scores are associated with the higher odds of metabolic syndrome in Romanian population. Our findings are in line with the above research findings that altered skinfold thickness may result in unfavourable HDL levels which may predispose to cardiometabolic disease risk. However, the age, gender and BMI were found to nullify the significance with higher age and BMI were associated with higher skinfold thickness rather than the HDL.

Our study found no significant association of other lipid variables (total cholesterol and triglycerides), glucose levels and BP with the skinfold thickness. Our study contrasts the findings of the earlier literature which found significant association between the lipid profile and skinfold thickness derived fat percentages [18]. High fasting glucose, triglycerides and cholesterol, though identified as positive CVD risk factor (detrimental) and are associated with excess peripheral fat [18, 22]. LDL and total cholesterol are associated with truncal and abdominal fat patterns. However, our study did not find any significant association. Further, we have not assessed the association of independent skinfold thickness with the biochemistry parameters. The participants of our cross-sectional study were young and healthy with no altered CVD risk markers. We recommend future trials to include older office workers or the clinical population, to substantiate the association of the triglycerides, cholesterol and blood glucose with peripheral fat percentage in segmental patterns (trunk, arm and legs).

The altered lipid profile may exhibit pleiotropic effects contributing to atherogenicity in blood vessels, impacting vascular dimensions and pressure [23]. VLDL, a key carrier of triglycerides and glucose to peripheral tissues for utilization, has been identified as a critical driver of atherogenic cardiovascular diseases [23, 24]. Few observational studies have established the association of skinfold thickness with the elevated BP [17, 21]. However, our study did not find a significant association of neither lipid markers such as total cholesterol, triglycerides nor BP with skinfold thickness-derived fat percentages among sedentary office workers. This may be the similar reasons cited above as our study participants were healthy with no established CVD.

Age and BMI were found to mediate the association of body fat percenatges with the biochemistry CVD risk markers [25]. The mediation effects of age and BMI has been found in the previous observational studies also. In primary care screening, age and BMI could be considered as mediators when considering skinfold thickness derived fat percentages.

### Strength and limitations

The main strength of the study is the homogeneity of the study population (sedentary office workers) conducted in low-middle income country where the cardiometabolic risks are surging. Further the study estimated the body fat percentages through inexpensive skinfold measurement whereas previous observational trials measured fat percentages through computerised tomography and air displacement plethysmography [26]. Hence our study is ecologically valid. Few limitations of the study are: (1) Although the sample size was estimated, we acknowledge that it may be insufficient for drawing definitive conclusions or generalizations. Our study specifically aimed to include a homogeneous sample of sedentary office workers; (2) as this is a cross-sectional study, causal inferences should be made with caution; (3) skinfold measured fat percentages though claimed to be valid, still depends upon the expertise and the regression equations from which the body density is derived. Since the present study relied on Western regression equations for body density estimation, the results may not be generalised. Indian studies exploring regression equations on skinfold derived fat percentage estimation specific to Indian population are warranted; (4) though age and BMI were added covariates, the present study did not control other potential confounders for the relation such as diet, sleep, smoking, alcoholism and psychological factors.

## Conclusions

Among the biochemical markers examined, only HDL demonstrated a negative association with skinfold thickness-derived fat percentage, while other CVD markers—such as total cholesterol, triglycerides, glucose, and blood pressure—showed no significant relationship. Age and BMI emerged as key mediating factors influencing both fat measurements and biochemical CVD markers. Given the cost-effectiveness of skinfold thickness-derived fat measurements, they could be incorporated into patient-centered care for CVD risk screening. Further research is warranted to assess the utility of these measurements in primary care settings and occupational health assessments.

## Data Availability

The datasets generated during and/or analysed during the current study are available from the corresponding author on reasonable request.

## Abbreviations

BMD: Body mass index
CVD: Cardiovascular diseases
DBP: Diastolic blood pressure
HDL: High density lipoprotein
MAP: Mean arterial pressure
SBP: Systolic blood pressure
